# Effect of the inclusion of extruded flaxseed in the diet of fattening pigs on lipid metabolism and tissue redox status

**DOI:** 10.1038/s41598-023-40378-0

**Published:** 2023-08-16

**Authors:** Anna Czech, Kamila Klimiuk, Iwona Sembratowicz

**Affiliations:** Department of Biochemistry and Toxicology, Faculty of Animal Sciences and Bioeconomy, University of Life Sciences in Lublin, Akademicka 13, 20-950 Lublin, Poland

**Keywords:** Lipids, Biochemistry, Physiology, Biomarkers

## Abstract

The aim of the study was to evaluate the effect of a diet containing extruded flaxseed (*Linum usitatissimum* L.) on the fatty acid composition of the loin, blood lipid parameters, and the redox status of tissues of finishing pigs. A total of 160 weaners (about 30–110 kg BW) were assigned to four experimental groups of 40 animals each (5 replicates with 8 individuals each). Group C (control) received a diet in which the fat source was soybean oil, while in groups 2FE, 4FE and 6FE soybean meal was replaced with extruded flaxseed in the amount of 2%, 4% or 6%, respectively. The diet containing extruded flaxseed reduced cholesterol levels in the blood plasma of pigs (grower 2FE and 6FE vs. C; finisher 2FE, 4FE and 6FE vs. C) and loin muscle (2FE, 4FE and 6FE vs. C). A decrease in the atherogenic LDL-C fraction and in the content of triacylglycerols was also noted in the blood plasma of grower and finisher pigs receiving flaxseed (2FE, 4FE and 6FE vs. C). The beneficial effects noted in the experimental pigs also included an increase in the overall content of n-3 PUFAs, especially ALA (18:3 n-3), and a reduction in the n-6/n-3 ratio. This was especially evident at 4% and 6% inclusion of flaxseed. The highest proportion of flax (6%) in the blend increased lipid peroxidation, as evidenced by the increase in the content of LOOH and MDA in the blood plasma of grower and finisher pigs. For this reason, a 4% share of flaxseed in the diet of fattening pigs seems to be optimal, while higher levels require an additional supply of exogenous antioxidants.

## Introduction

Pork has been highly popular among inhabitants of Europe for years, but consumers are increasingly interested in its nutritional and health quality, especially the content and quality of its fat. Taking into account health reasons, especially the risk of cardiovascular disease, the most desirable is meat containing less saturated fatty acids (SFA) and cholesterol, and more unsaturated fatty acids (UFA), especially polyunsaturated fatty acids (PUFA) from the n-3 family^1^. Dietary n-3 PUFAs have been shown to beneficially influence not only the circulatory system, but also immune processes, nervous system function, and the course of inflammatory reactions^2–5^. In monogastric animals, it is fairly easy to modify the composition of fatty acids in the muscles and the adipose tissue, especially the PUFA content, by means of diet. A variety of supplements are used to enrich pork meat with valuable n-3 fatty acids; these can be vegetable oils (e.g. hemp or rapeseed), microalgae, or fish oil^6–8^. There is also great interest in the use of various flaxseed products in the diet of pigs^9–12^. Flax is a valuable oilseed plant with very high (about 54%) content of alpha-linolenic acid (ALA), representing the n-3 PUFA family^13^. Flaxseeds are also a source of many active compounds with health-promoting properties, including polyphenolic substances, especially lignans, high-quality protein, and soluble fiber, as well as vitamins and minerals^14,15^. This rich chemical composition of flaxseeds is responsible for their multifaceted biological activity. They have been shown to have a beneficial effect on various metabolic processes, including lipid and carbohydrate metabolism, inflammatory reactions, and gastrointestinal function^16^. The inclusion of flaxseeds in the diet of pigs may therefore not only enrich the tissues with valuable n-3 PUFAs, but may also improve the animals’ overall health condition. However, their high content of ALA, an n-3 PUFA, entails a certain risk associated with potential intensification of oxidative reactions in the body. This is due to the high susceptibility of these acids to oxidation, which increases with the number of double bonds in the molecule^17^. Oxidative changes in meat lipids lead to deterioration of its physicochemical and organoleptic properties and its storage quality^18^. Moreover, the products generated in this process (hydroperoxides, aldehydes, and cholesterol oxides) can have harmful effects on consumer health^18^.

In view of the above, choosing the appropriate inclusion level of a feed component with high PUFA content, such as extruded flaxseed, is essential to obtaining a product (meat) with good physicochemical parameters, i.e. a favorable fatty acid composition and a reduced amount of lipid peroxidation products. At the same time, monitoring of indicators of lipid metabolism (levels of CHOL, TG, LDL-C, and HDL-C in the blood plasma) and antioxidant parameters in the blood during different stages of fattening (Grower and Finisher) makes it possible to assess the reaction of the body to products rich in PUFAs.

Therefore the aim of the study was to determine the effect of extruded flaxseed in the amount of 2%, 4% and 6% of diets for grower and finisher pigs on lipid metabolism and tissue redox status.

## Results

The plasma of grower pigs receiving 6% or 2% extruded flaxseed (groups 6FE and 2FE, respectively) had significantly lower cholesterol content than that of pigs from the control group (C group). The CHOL (total cholesterol) concentration in the plasma of finisher pigs was significantly lower in groups 6FE, 4FE and 2FE than in group C (*p* < 0.001), and the dose dependence was linear (*p* = 0.004) (Table 1). The HDL-C percentage in the plasma of grower and finisher pigs receiving 2%, 4% and 6% extruded flaxseed was significantly higher than in group C (*p* = 0.018 and *p* = 0.044, for grower and finisher, respectively). The plasma of grower and finisher pigs receiving a diet with extruded flaxseed had significantly lower TG (*p* = 0.042 and *p* < 0.001, respectively) and LDL-C levels (*p* = 0.006 and *p* = 0.004, respectively) compared to the control group (Table 1).

**Table 1:** Lipid profile parameters in the plasma of fattening pigs.

Treatment^1^	CHOL	CHOL	HDL-C	HDL-C	%HDL-C	%HDL-C	TG	TG	LDL-C	LDL-C	CHOL/HDL-C	CHOL/HDL-C
	Grower	Finisher	Grower	Finisher	Grower	Finisher	Grower	Finisher	Grower	Finisher	Grower	Finisher
	mmol l^−1^		mmol l^−1^				mmol l^−1^		mmol l^−1^			
C	2.09^a^	2.49^a^	1.22	1.41^a^	58.53^b^	56.73^c^	0.668^a^	0.991^a^	0.567^a^	0.630^a^	1.71^a^	1.56
2FE	1.80^b^	2.31^b^	1.18	1.41^a^	65.75^a^	61.16^a^	0.615^b^	0.770^b^	0.339^c^	0.549^b^	1.53^b^	1.57
4FE	1.93^ab^	2.13^c^	1.25	1.32^a^	64.71^a^	62.21^a^	0.582^b^	0.704^c^	0.419^b^	0.489^b^	1.55^b^	1.45
6FE	1.83^b^	2.02^d^	1.22	1.18^b^	66.85^a^	58.28^b^	0.614^b^	0.608^d^	0.328^c^	0.565^b^	1.50^b^	1.47
SEM^2^	0.028	0.047	0.014	0.028	0.885	0.872	0.009	0.030	0.024	0.030	0.022	0.028
*P* value												
Diet^3^	0.001	< 0.001	0.586	0.011	0.018	0.044	0.042	< 0.001	0.006	0.004	0.009	0.526
Linear FE^4^	0.069	0.004	0.227	0.050	0.747	0.064	0.274	0.037	0.400	0.348	0.373	0.360
Quadratic FE^4^	0.188	0.239	0.607	0.066	0.081	0.037	0.087	0.193	0.033	0.054	0.986	0.859

^a–d^Means with different superscripts are statistically different across all 4 treatments (*p* < 0.05).

CHOL, total cholesterol; TG, triacylglycerols; HDL-C, high-density lipoprotein cholesterol; LDL-C, low-density lipoprotein cholesterol.

^1^There were 4 dietary treatments. The first diet (control; C) without additive extruded flaxseed. Diets 2–4 contained 2% (2FE), 4% (4FE) and 6% (6FE) extruded flaxseed.

^2^SEM—standard error of the mean.

^3^*P* value for overall effect of dietary treatment (diets 1–4).

^4^Orthogonal polynomial (linear FE and quadratic FE) contrasts were performed to test the effect of the extruded flaxseed level in the diets.

SOD (superoxide dismutase) activity in the plasma of grower and finisher pigs receiving 6% FE in their diet (6FE group) was significantly higher than in group C (*p* < 0.001 and *p* = 0.008, respectively), but also in comparison to groups 2FE and 4FE; this relationship was linear (*p* < 0.001 and *p* = 0.049, respectively) (Table 2). CAT (catalase) activity in the plasma of grower pigs from group 6FE was also significantly higher than in the other experimental groups (*p* < 0.001). The statistical analysis showed that the level of flaxseed significantly influenced SOD and CAT activity, and that this was a linear relationship, i.e. the activity of the enzymes increased with the share of flaxseed (for SOD *p* < 0.001 and *p* = 0.049 for grower and finisher pigs, respectively; for CAT *p* < 0.001).

**Table 2:** Redox parameters in the plasma of fattening pigs.

Treatment^1^	SOD	SOD	CAT	CAT	Vitamin C	Vitamin C	FRAP	FRAP	LOOH	LOOH	MDA	MDA
	Grower	Finisher	Grower	Finisher	Grower	Finisher	Grower	Finisher	Grower	Finisher	Grower	Finisher
	U ml^−1^		U ml^−1^		mg l^−1^		µmol l^−1^		µmol l^−1^		µmol l^−1^	
C	43.03^c^	36.18^c^	3.81^b^	3.74	0.22	0.17^b^	51.19^b^	38.85^c^	4.26^b^	5.29^b^	4.02^b^	4.12^b^
2FE	43.49^c^	41.70^b^	3.94^b^	3.80	0.24	0.21^a^	62.64^a^	41.74^bc^	5.46^a^	4.98^c^	4.32^ab^	4.28^b^
4FE	50.52^b^	41.95^b^	4.07^b^	3.71	0.23	0.21^a^	64.88^a^	44.34^b^	5.38^a^	5.14^bc^	4.48^ab^	4.65^ab^
6FE	52.58^a^	44.67^a^	5.84^a^	4.10	0.25	0.22^a^	64.91^a^	47.13^a^	5.88^a^	6.37^a^	5.00^a^	5.10^a^
SEM^2^	0.892	0.800	0.168	0.113	0.007	0.003	1.512	0.853	0.145	0.102	0.095	0.096
*P*-value												
Diet ^3^	< 0.001	0.008	< 0.001	0.720	0.648	< 0.001	0.008	0.020	< 0.001	< 0.001	0.008	0.004
Linear FE^4^	< 0.001	0.049	< 0.001	0.482	0.652	0.265	0.607	0.030	0.094	< 0.001	0.003	0.008
Quadratic FE^4^	0.232	0.489	0.064	0.528	0.432	0.888	0.771	0.963	0.174	< 0.001	0.310	0.872

^a–d^Means with different superscripts are statistically different across all 4 treatments (*p* < 0.05).

SOD, superoxide dismutase; CAT, catalase; FRAP, the total antioxidant potential of the plasma; LOOH, lipid hydroperoxide; MDA, malondialdehyde.

^1-4^see Table 1.

Vitamin C content in the plasma of finisher pigs receiving a diet with extruded flaxseed (groups 6FE, 4FE and 2FE) was significantly higher than in group C (*p* < 0.001). The FRAP (ferric reducing antioxidant power) value and LOOH (lipid hydroperoxide) content in the plasma of grower pigs fed flaxseed was significantly higher than in the other experimental groups (*p* = 0.008 and *p* < 0.001, respectively). The plasma of finisher pigs receiving 6% EF in the diet had a significantly higher FRAP value and LOOH content compared to the other experimental groups (*p* = 0.020 and *p* < 0.001, respectively). The statistical analysis showed that the level of flaxseed significantly influenced the FRAP value and LOOH level in the finisher pigs and the MDA level in the grower and finisher pigs. This was a linear relationship, i.e. the value of these parameters increased with the share of flaxseed (Table 2).

In pigs receiving a diet with 4% FE, SOD and vitamin C content in the *longissimus dorsi* muscle were significantly higher than in the control group (*p* = 0.046 and *p* = 0.011, respectively) (Table 3). CAT activity in the *longissimus dorsi* muscle was also significantly higher in the group receiving 4% FE, as well as in the group receiving 6% FE (groups 4FE and 6FE), compared to the other experimental groups (*p* < 0.001) (Table 3). The inclusion of extruded flaxseed reduced the level of LOOH relative to group C (*p* < 0.001) and also reduced the MDA level in the *longissimus dorsi* muscle relative to group C (*p* < 0.001).

**Table 3:** Redox parameters in the *longissimus dorsi* muscle.

Treatment^1^	SOD	CAT	Vitamin C	LOOH	MDA
	U mg protein^−1^	U mg protein^−1^	mg g^−1^	µmol mg^−1^	nmol mg^−1^
C	26.43^b^	3.74^b^	0.203^b^	5.40^a^	0.494^a^
2FE	26.77^b^	3.05^c^	0.251^ab^	4.01^b^	0.297^bc^
4FE	28.33^a^	4.35^a^	0.301^a^	3.82^b^	0.260^c^
6FE	26.28^b^	4.90^a^	0.281^ab^	4.31^b^	0.354^b^
SEM^2^	0.297	0.166	0.011	0.135	0.021
*P* value					
Diet ^3^	0.046	< 0.001	0.021	< 0.001	< 0.001
Linear FE^4^	0.484	< 0.001	0.362	0.133	0.003
Quadratic FE^4^	0.013	0.108	0.229	0.054	< 0.001

^a–d^Means with different superscripts are statistically different across all 4 treatments (*p* < 0.05).

SOD, superoxide dismutase; CAT, catalase; LOOH, lipid hydroperoxide; MDA, malondialdehyde.

^1^^–^^4^ see Table 1.

The content of SFAs in the *longissimus dorsi* muscle of the pigs from groups 2FE and 4FE was significantly lower (*p* < 0.001) than in the control group and group 6FE (*p* < 0.001) (Table 4). This corresponded to a significantly lower level of 16:0 in group 2FE than in groups C and 6FE (*p* < 0.001) and significantly lower levels of acids 18:0 and 20:0 in the *longissimus dorsi* muscle of pigs from group 4FE compared to group C (*p* < 0.001, *p* = 0.020, respectively) (Table 5).

**Table 4:** Fatty acid profile of total lipids in the *longissimus dorsi* muscle.

Treatment^1^	Individual SFA (g/100 g of TFA)				Individual MUFA (g/100 g of TFA)				Individual PUFA (g/100 g of TFA)						
	14:0	16:0	18:0	20:0	16:1 n-9	18:1 n-9	18:1 n-7	20:1 n-9	18:2 n-6	22:2	18:3 n-3	20:3 n-3	20:4 n-6	Other	
C	2.98	22.84^a^	12.73^a^	0.408^a^	3.71	43.28	5.07^a^	0.033^c^	5.03	0.293^ab^	0.575^b^	0.678^b^	0.513^a^	1.86^b^	
2FE	2.62	21.62^b^	12.42^ab^	0.371^ab^	3.62	43.06	4.44^b^	0.059^b^	4.72	0.240^b^	0.986^a^	0.875^a^	0.380^b^	4.59^a^	
4FE	2.55	22.31^ab^	11.99^b^	0.353^b^	3.65	42.95	4.10^b^	0.063^b^	4.90	0.290^ab^	1.00^a^	0.945^a^	0.318^c^	4.59^a^	
6FE	2.79	22.73^a^	12.71^a^	0.340^b^	3.55	43.36	4.92^a^	0.146^a^	4.95	0.315^a^	1.21^a^	0.788^ab^	0.388^b^	1.81^b^	
SEM^2^	0.050	0.133	0.089	0.007	0.026	0.054	0.083	0.020	0.087	0.007	0.050	0.023	0.015	0.301	
*P value*															
Diet^3^	0.099	< 0.001	< 0.001	0.011	0.361	0.067	< 0.001	0.042	0.061	0.001	< 0.001	< 0.001	< 0.001	< 0.001	
Linear FE^4^	0.134	0.021	0.093	0.066	0.112	0.089	0.055	0.027	0.088	< 0.001	0.086	0.085	0.078	0.053	
Quadratic FE^4^	0.149	0.069	0.042	0.095	0.685	0.337	0.051	0.540	0.026	0.062	0.091	0.006	0.053	0.001	

^a–d^Means with different superscripts are statistically different across all 4 treatments (*p* < 0.05).

SFA, saturated fatty acid; MUFA, monounsaturated fatty acid; PUFA, polyunsaturated fatty acid; TFA, total fatty acid.

^1^^–^^4^ see Table 1.

**Table 5:** Fatty acid profile of total lipids in the *longissimus dorsi* muscle.

Treatment^1^	SFA	MUFA	PUFA	n-3	n-6	n-6/n-3	n-3/n-6	AI	TI	h/H	CHOL (mg/100 g)
C	38.96^a^	52.09^a^	7.09^b^	1.25^c^	5.55^a^	4.42^a^	0.226^b^	0.590	1.18^a^	2.14	124.3^a^
2FE	37.04^b^	51.17^ab^	7.20^ab^	1.86^a^	5.10^b^	2.74^b^	0.365^a^	0.561	1.08^b^	2.25	102.9^b^
4FE	37.20^b^	50.76^b^	7.45^ab^	1.95^a^	5.22^b^	2.68^b^	0.373^a^	0.552	1.08^b^	2.18	106.3^b^
6FE	38.56^a^	51.98^ab^	7.65^a^	2.00^a^	5.33^ab^	2.67^b^	0.374^a^	0.571	1.09^b^	2.18	95.50^b^
SEM^2^	0.228	0.122	0.034	0.070	0.045	0.185	0.015	0.004	0.012	0.012	2.84
*P* value											
Diet^3^	< 0.001	< 0.001	0.038	< 0.001	< 0.001	< 0.001	< 0.001	0.062	< 0.001	0.088	0.001
Linear FE^4^	0.023	0.104	0.046	< 0.001	0.011	0.073	0.161	0.087	0.154	0.061	0.129
Quadratic FE^4^	0.088	0.101	0.097	0.127	0.059	0.094	0.223	0.084	0.230	0.099	0.044

^a–d^Means with different superscripts are statistically different across all 4 treatments (*p* < 0.05).

SFA, saturated fatty acid; MUFA, monounsaturated fatty acid; PUFA, polyunsaturated fatty acid; AI, atherogenic index; TI, thrombogenic index; h/H, hypocholesterolemic/hypercholesterolemic ratio.

^1^^–^^4^ see Table 1.

MUFA content was significantly lower in the *longissimus dorsi* muscle of pigs from group 4FE compared to the control group (*p* < 0.001) (Table 4). The *longissimus dorsi* muscle of pigs from groups 2FE and 4FE had significantly lower levels of acids 18:1 and 20:1 compared to groups C and 6FE (*p* < 0.001). The content of 20:1 was significantly higher in groups 2FE and 4FE than in group C, but significantly lower than in group 6FE (Table 4).

Significantly higher PUFA content was noted in the muscle of pigs from 6FE compared to the control. The content of n-3 acids was significantly higher in groups 6FE, 4FE and 2FE compared to group C (*p* < 0.001); this relationship was linear (*p* < 0.001). This corresponded to significantly higher levels of 18:3 and 20:3 acids in the groups receiving a diet with extruded flaxseed compared to group C (*p* < 0.001) (Table 4).

The levels of n-6 were significantly higher in the control group than in groups 2FE and 4FE (Table 5). In all groups of pigs receiving a diet with extruded flaxseed, the 20:4 (n-6) level was significantly higher than in the control group (*p* < 0.001; Table 4).

The n-6/n-3 ratio was significantly and the n-3/n-6 ratio was significantly higher in the *longissimus dorsi* muscle of pigs from groups receiving a diet with extruded flaxseed (*p* < 0.001) (Table 5).

The TI value and cholesterol content in the *longissimus dorsi* muscle was significantly lower in pigs from groups 2FE, 4FE and 6FE than in the control (*p* < 0.001 and *p* = 0.001, respectively). The extruded flaxseed level significantly influenced the SFA, PUFA, n-3 and n-6 values in the *longissimus dorsi* muscle; this was a linear relationship, i.e. the value of these parameters increased with the FE level (*p* = 0.023, *p* = 0.046, *p* < 0.001 and *p* = 0.011, respectively) (Table 5).

## Discussion

The results of an extensive meta-analysis of the biological effects of flaxseed indicate that a flaxseed diet significantly reduces total and LDL cholesterol in the blood, while its effect on TG and HDL-C is minor^19^. In our study, the inclusion of extruded flaxseed in the diet of pigs modified all lipid parameters, decreasing the levels of LDL-C, CHOL and TG while increasing that of HDL-C. These differences were noted even at the lowest inclusion level, i.e. 2% of the diet. According to Clark et al.^20^, the hypolipidemic effects of lower levels of flaxseed may be greater than the effects of higher levels. Song et al.^11^, who used 3% or 1.5% flaxseed in the diet of pigs, found that it did not affect LDL-C, HDL-C and TG, but only reduced the total cholesterol level. Liu and Kim ^21^ reported that the reduction in the n-6/n-3 ratio from 15:1 to 5:1 resulting from the inclusion of flaxseed oil in the diet of pigs caused a significant decrease in the levels of TG, total cholesterol, and LDL cholesterol. The effect of flaxseed on lipid metabolism, observed in the present study and in those of other authors, is in part due to its high content of alpha-linolenic acid. Ukropec et al.^22^, who analyzed the effect of fish oils on blood lipid parameters in rats, reported that the hypolipidemic effect of n-3 PUFAs is due to the intensification of β-oxidation processes, especially in the liver, and to a reduction in leptin expression. Leptin, produced mainly in adipose tissue, is a pleiotropic cytokine which influences lipogenesis and lipolysis as well as satiety^23^. The molecular basis for the effects of PUFAs on lipid metabolism is associated with modulation of the activity of certain transcription factors, such as PPARs (peroxisome proliferator activated receptors) and SREBP-1c- (sterol regulatory element binding protein 1c)^24^. It should be noted that besides ALA, other components of flaxseed, such as lignans or soluble fibre, can modify blood lipid parameters, especially the content of cholesterol and its fractions^25,26^.

### Effect on fatty acid composition

The fatty acid profile in the tissues of monogastric animals is determined to a greater extent by the diet^27^. Therefore it is quite possible to obtain meat and fat that is more valuable in this respect, and in particular to enrich them with PUFAs, which cannot be synthesized in the body but must be supplied with feed. As flaxseeds have high concentrations of ALA (52–58%), their use as a component of pig diets can have measurable benefits in the form of increased levels of this acid in the tissues^12,28,29^. ALA ingested with feed can be directly incorporated into the tissues or further metabolized to other n-3 acids, i.e. EPA (eicosapentaenoic acid) or DHA (docosahexaenoic acid), of which it is a precursor^24^. In pigs, however, as in humans, this conversion is limited^30^. In the present study, a 6% share of flaxseed led to a significant increase in total PUFA content, as a result of the increased content of 18:3 n-3 and 20:3 n-3. Most likely due to low intake of linoleic acid C18:2 n-6 (LA), there was a significant decrease in the content of arachidonic acid (ARA) 20:4 n-6, of which LA is the main precursor. Similar observations, i.e. a decrease in ARA, have been made in other experiments using flaxseed^28,31^. Owing to the significant reduction in the content of 20:4 n-6 and the increase in 18:3 n-3 and 20:3n-3, there was a beneficial decrease in the n-6/n-3 PUFA ratio. An increase in n-3 PUFA in the diet is very important, because consumption of n-6 acids is generally much higher, so that the n-6/n-3 PUFA ratio significantly deviates from the recommended value 1:1, although, due to eating habits and genetic changes, this ratio is higher^3^. Omega-6 acids are a source of pro-inflammatory eicosanoids, whereas omega-3 acids are precursors of compounds with the opposite effect, positively influencing numerous processes in the body. In our experiment, this ratio decreased about 1.6-fold and was essentially independent of the level of flaxseed used. Other researchers^10,29^ have succeeded in reducing this ratio 2–threefold, while ratios in the groups not receiving the flaxseed diet were higher than in our experiment. The positive effects of the use of a diet containing 2% and 4% flaxseed also included a decrease in the total SFA content, and at 4% the total MUFA content decreased as well. Similarly, Okrouhlá et al.^28^ and Bečková and Václavková^33^ reported that flaxseed supplementation caused a decrease in the total MUFA content in the meat of pigs, while Tarricone et al.^32^ noted a decrease in total SFA. Given the negative impact of high intake of saturated fatty acids on blood lipid parameters and the increased risk of cardiovascular disease, this is a highly desirable phenomenon. The health value of meat, specifically the potential relationship between its consumption and the occurrence of atherosclerosis or platelet aggregation, is indicated by indexes: atherogenic (AI) and thrombogenic (TI). The decline in their values is associated with a decrease in the content of saturated acids with atherogenic (C:12, C:14, and C:16) or thrombogenic (C:14, C:16, and C:18) effects^34^. The results of our experiment showed that irrespective of the level of flaxseed in the diet, the thrombogenic index decreased significantly, which was not observed in the case of the atherogenic index. These results are consistent with the findings of other authors, who used 25 and 50 g/kg linseed oil sediment^10^ and 150 g/kg crushed flaxseed Okrouhlá et al.^28^ and Čítek et al.^31^. A decrease in both indexes, i.e. TI and AI, was reported by Tarricone et al.^32^, who included 3% extruded flaxseed in the diet of Nero Lucano pigs.

Apart from its effect on the fatty acid profile, the diet modification caused a significant reduction in the cholesterol content of the loin muscle, which should also be considered beneficial for human health. There are indications that increasing the share of n-3 PUFA in the diet by introducing linseed or fish oil may result in lowering the amount of cholesterol in the tissues of slaughter animals^35,36^.

### Redox status

The balance between oxidation and reduction processes in the body is maintained by well-functioning antioxidant mechanisms. These include both enzymes, such as SOD (superoxide dismutase), CAT (catalase) and GPx (glutathione peroxidase), and non-enzymatic antioxidants^37^. Intensification of oxidative processes and redox imbalance, i.e. oxidative stress, can result from increased intake of PUFAs, especially n-3 acids, which are highly susceptible to oxidation^38^. The most commonly used marker of meat lipid peroxidation is malondialdehyde (MDA), which is relatively stable compound that can achieve a fairly high concentration in food^39^. In the present study, the inclusion of flaxseed (especially at 6%) in the diet of pigs resulted in intensification of oxidative reactions, as indicated by the elevated levels of lipid peroxidation products, i.e. LOOH and MDA, in the blood of the experimental pigs. However, neither MDA nor LOOH increased in the loin of pigs on a flaxseed diet, but decreased, which is difficult to explain. Since these compounds are formed as a result of oxidation of PUFAs, their content in the muscles should be correlated with the levels of these acids. Marcinčák et al.^40^ reported that significant oxidative changes in the meat of pigs receiving flaxseed in their diet took place just 24 h after slaughter and increased with storage time. A study using 5% and 10% inclusion of flaxseed in the diet of fattening pigs for 3 or 6 weeks showed that the increase in PUFA in the muscles was accompanied by an increase in the MDA level^12^. However, 5% supplementation of flaxseed for 3 weeks had no negative effect on the oxidative stability of the meat, despite some increase in n-3 PUFAs. It should be noted that there are some indications that n-3 PUFAs exhibit rather antioxidant and not pro-oxidant activity, positively influencing the redox status of the body^41^. This effect may be caused by induction of a signaling pathway associated with nuclear factor erythroid-derived 2-like 2 (Nrf2), whose basic function is to activate genes encoding antioxidant enzymes, which are the most important elements of antioxidant defense^42^. Our experiment showed an increase in the activity of antioxidant enzymes SOD and CAT in both the loin muscle and the plasma, with the smallest effects noted in the case of the lowest inclusion of flaxseed. As a result, the intensity of lipid peroxidation measured by the plasma MDA level was reduced. It is worth noting that the inclusion of flaxseed in the diet not only increases intake of n-3 PUFAs, but also provides many recognized exogenous antioxidants, particularly lignans. One of the major lignans in flax, SDG (secoisolariciresinol diglucoside), has been found to have a high capacity to scavenge hydroxyl radicals, and thus to protect PUFAs against oxidation^43^. Flaxseed is also a source of other powerful antioxidants, such as tocopherols and phenolic acids^44^. These exogenous antioxidants can significantly increase the antioxidant potential of the plasma, which was demonstrated in our study, as the groups receiving flaxseed showed an increase in the FRAP value. There was also an increase in the content of vitamin C in both the blood and the loin. An extensive meta-analysis of the properties of flaxseed oil included in the human diet showed that it is effective at counteracting oxidative stress, as indicated by an increase in total antioxidant capacity and significantly reduced blood MDA levels^45^.

## Conclusions

The inclusion of extruded flaxseed in the diet of pigs had clear positive effects on lipid metabolism, resulting in a reduced cholesterol level in both the blood plasma and the *longissimus dorsi* muscle. The presence of extruded flaxseed in the diet also increased the level of PUFAs, especially 18:3 (n-3), resulting in a reduction in the n-6/n-3 ratio, which is highly beneficial to consumers. The higher inclusion levels, i.e. 4% and 6%, were most effective in this regard. However, the use of the higher 6% level of extruded flaxseed caused an increase in lipid peroxidation end products, i.e. MDA and LOOH, which may indicate an increase in oxidation processes. Therefore the optimum level of extruded flaxseed in diets for fattening pigs seems to be 4%.

## Material and methods

The experiment was conducted on 160 weaners (Polish Large White x Neckar) assigned to four experimental groups. Each group consisted of 40 animals, which were kept in 5 pens with 8 animals per pen. The pigs were tagged and weighed before being assigned to groups. Each group contained an equal number of gilts and barrows. Variation in body weight within and between replicates (pens) was minimized as far as practically possible. The experiment was begun when the weaners weighed about 25 kg and was completed when they reached slaughter weight, i.e. about 115 kg.

The experimental factor was extruded flaxseed. The animals in the experimental groups received diets with 2% (group 2FE), 4% (group 4FE), or 6% (group 6% FE) extruded flaxseed in place of soybean meal. Soybean oil was added to the experimental diets to equalize the level of fat. A control group (group C) was created as well, in which the animals received a diet with soybean oil as the fat source. The diets were composed of wheat, rapeseed meal, soybean meal, barley, wheat bran, soybean oil, dicalcium phosphate, calcium, L-lysine chloride, and a mineral and vitamin premix (2% of the grower diet—about 25–70 kg BW; 1.5% of the finisher diet—about 71–115 kg BW). The mineral and vitamin premix contained (in 1 kg) vitamin A 600,000 IU, vitamin D_3_ 60,000 IU, vitamin E 3,000 mg, vitamin K_3_ 120 mg, vitamin B_1_ 120 mg, vitamin B_2_ 240 mg, vitamin B_6_ 240 mg, nicotinic acid 1,600 mg, pantothenic acid 800 mg, folic acid 160 mg, biotin 10 mg, vitamin B_12_ 1.6 mg, choline chloride 12 g, Mg 0.8 g, Fe 6 g, Zn 5.6 g, Mn 2.4 g, Cu 6.4 g, J 40 mg, Se 16 mg, and Co 16 mg. The exact ingredient composition and chemical composition of the diet have been published in a paper by Klimiuk et al.^46^.

The grower diet contained about 5.4% crude fat, about 17.0% crude protein, 4.80% crude fibre, 1.40% lysine, about 6.45% methionine + cysteine, 0.65% phosphorus, and 0.71% calcium. Metabolic energy was 13.0 MJ/kg.

The finisher diet contained about 6.0% crude fat, about 15.0% crude protein, about 6.0% crude fibre, 0.90% lysine, about 5.8%, methionine + cysteine, 0.55% phosphorus, and 0.66% calcium. Metabolic energy was 12.5 MJ/kg.

The content of nutrients, amino acids, phosphorus, and calcium in all groups met NRC requirements for pigs^47^. They received feed and water ad libitum throughout the study.

### Experimental procedures

The experimental procedures used throughout this study were approved by the Second Local Ethics Committee on Animal Experimentation of the University of Life Sciences in Lublin, Poland.

The study was carried out in compliance with the ARRIVE guidelines.

All methods were carried out in accordance with relevant guidelines and regulations regarding to life animal studies and the procedures complied with the Directive 2007/526/EC of the European Parliament and of the Council on the protection of animals used for scientific purposes.

Blood for analysis was collected at the end of each stage of the experiment (at ~ 70 kg and ~ 110 kg). Blood was sampled from 24 pigs (6 from each group). The animals had no access to feed for 12 h before sampling. Blood was drawn from the jugular vein into 10 ml heparinized test tubes.

After final weighing (~ 110 kg) during slaughter, samples of the *longissimus dorsi* muscle were taken from the same animals from which blood was drawn (n = 6) in each experimental group, for analysis of lipid and redox parameters.

### Laboratory analyses

#### Biochemical analyses

Plasma was obtained by centrifuging whole blood at 3,500 rpm for 15 min in a laboratory centrifuge and stored in Eppendorf tubes at − 80 °C until analysis.

Cormay tests were used for spectrophotometric determination of selected biochemical parameters in the blood plasma: total cholesterol (CHOL), triacylglycerols (TG), and high-density lipoprotein cholesterol (HDL-C). Low-density lipoprotein cholesterol (LDL-C) was calculated using the Friedewald equation^48^: LDL-C (mmol l^−1^) = CHOL–HDL-C—TG/2.2.

Concentrations of antioxidant enzymes superoxide dismutase (SOD) and catalase (CAT), as well as antioxidant system parameters, i.e. total antioxidant potential as ferric-reducing ability of plasma (FRAP), vitamin C content, and levels of lipid peroxidation products—peroxides (LOOH) and malondialdehyde (MDA), were determined in the plasma and tissues according to methods described in a previous paper Czech et al.^49^.

The content of fatty acids and cholesterol was analyzed following extraction of fat with a mixture of chloroform/methanol according to Folch et al.^50^. Further procedures were carried out according to standards PN-EN ISO 5509:2001^51^ and PN-EN ISO 5508:1996^52^.

Fatty acids were determined by gas chromatography in a Varian CP-3800 chromatograph. The operating conditions of the chromatograph for separation of fatty acids were as follows: a 0.25 mm CP WAX 52CB DF capillary column 60 m in length, gas carrier—helium, flow—1.4 ml/min, column temperature 120ºC gradually increased by 2ºC/min, analysis time 127 min, injector temperature 160ºC, detector temperature 160ºC, other gases—hydrogen and oxygen.

The content of fatty acids in the fat of the *longissimus dorsi* muscle was calculated according to Weihrauch et al.^53^. Parameters of lipid quality, i.e. the atherogenic index (AI) and thrombogenic index (TI), were calculated according to the following equations^54^:

$${\text{AI}}\, = \,[\left( {{4}\, \times \,{\text{C14}}:0} \right)\, + \,{\text{C16}}:0\left] / \right[{\text{n}} - {\text{6 PUFA}}\, + \,{\text{n}} - {\text{3 PUFA}}\, + \,{\text{MUFA}}].$$

$${\text{TI}}\, = \,[{\text{C14}}:0\, + \,{\text{C16}}:0\, + \,{\text{C18}}:0\left] / \right[\left( {0.{5}\, \times \,{\text{MUFA}}} \right)\, + \,\left( {0.{5}\, \times \,{\text{n}} - {\text{6PUFA}}} \right)\, + \,\left( {{3}\, \times \,{\text{n}} - {\text{3 PUFA}}} \right)\, + \,{\text{n}} - {3}/{\text{n}} - {\text{6 PUFA}}].$$

The hypocholesterolemic/hypercholesterolemic ratio (h/H) was obtained according to Fernández et al.^55^.

$${\text{h}}/{\text{H}}\, = \,\left( {{\text{C18}}:{1}\, + \,{\text{C18}}:{2}\, + \,{\text{C18}}:{3}\, + \,{\text{C2}}0:{3}\, + \,{\text{C2}}0:{4}\, + \,{\text{C2}}0:{5}\, + \,{\text{C22}}:{4}\, + \,{\text{C22}}:{5}\, + \,{\text{C22}}:{6}} \right)/({\text{C14}}:0\, + \,{\text{C16}}:0).$$

## Statistical analysis

All data are expressed as means and SEM (standard error of the mean). The normality of data distribution was tested using the Shapiro–Wilk test, and equality of variance was tested by Levene’s test. Treatment means were compared by Tukey’s HSD (honest significant difference) test using Statistica 10 (Dell Software Inc., USA)^56^. Orthogonal polynomial contrasts were performed on the three treatments with extruded flaxseed (2FE, 4FE and 6FE) to test the linear and quadratic effects of flaxseed level on selected response variables. For all tests, an α level of *p* < 0.05 was used to determine statistical significance.

## Data Availability

The datasets used and/or analyzed during the current study available from the corresponding author on reasonable request. Correspondence and requests for materials should be addressed to A.C., K.K., or I.S.

## References

[1] Salter M (2013). Dietary fatty acids and cardiovascular disease. Animal.

[2] Calder PC, Yaqoob P (2009). Understanding omega-3 polyunsaturated fatty acids. Postgrad. Med..

[3] Simopoulos AP (2006). Evolutionary aspects of diet, the omega-6/omega-3 ratio and genetic variation: Nutritional implications for chronic diseases. Biomed. Pharmacother..

[4] Stanley JC (2007). UK food standards agency workshop report: The effects of the dietary n-6:n-3 fatty acid ratio on cardiovascular health. Br. J. Nutr..

[5] Calder P (2013). N-3 Fatty acids, inflammation and immunity: New mechanisms to explain old actions. Proc. Nutr. Soc..

[6] Mourot J, Guillevic M (2015). Effect of introducing hemp oil into feed on the nutritional quality of pig meat. OCL.

[7] de Tonnac A, Guillevic M, Mourot J (2018). Fatty acid composition of several muscles and adipose tissues of pigs fed n-3 PUFA rich diets. Meat Sci..

[8] Komprda T (2021). Fatty acid composition, oxidative stability, and sensory evaluation of the sausages produced from the meat of pigs fed a diet enriched with 8% of fish oil. J. Food Sci..

[9] Juárez M (2010). Feeding co-extruded flaxseed to pigs: Effects of duration and feeding level on growth performance and backfat fatty acid composition of grower–finisher pigs. Meat Sci..

[10] Leikus R (2018). Effect of linseed oil sediment in the diet of pigs on the growth performance and fatty acid profile of meat. Rev. Bras. Zootech..

[11] Song CH (2020). The ratio of dietary n-3 polyunsaturated fatty acids influences the fat composition and lipogenic enzyme activity in adipose tissue of growing pigs. Food Sci. Anim. Resour..

[12] Bartkovský M (2022). Effect of concentration of flaxseed (*Linum usitatissimum*) and duration of administration on fatty acid profile, and oxidative stability of pork meat. Animals.

[13] Silska G, Walkowiak M (2019). Comparative analysis of fatty acid composition in 84 accessions of flax (*Linum usitatissimum* L.). J. Pre-Clin. Clin. Res..

[14] Bernacchia R, Preti R, Vinci G (2014). Chemical composition and health benefits of flaxseed. Austin J. Nutr. Food Sci..

[15] Czech A, Ognik K, Klebaniuk R, Stępniowska A (2015). Chemical characteristic of the feed mixtures containing raw and extruded flax seeds. Przem. Chem..

[16] Goyal A, Sharma V, Upadhyay N, Gill S, Sihag M (2014). Flax and flaxseed oil: An ancient medicine & modern functional food. J. Food Sci Technol..

[17] Lyberg A, Fasoli E, Adlercreutz P (2005). Monitoring the oxidation of docosahexaenoic acid in lipids. Lipids.

[18] Huang X, Ahn DU (2019). Lipid oxidation and its implications to meat quality and human health. Food Sci. Biotechnol..

[19] Pan A, Yu D, Demark-Wahnefried W, Franco OH, Lin X (2009). Meta-analysis of the effects of flaxseed interventions on blood lipids. Am. J. Clin. Nutr..

[20] Clark WF (1995). Flaxseed: A potential treatment for lupus nephritis. Kidney Int..

[21] Liu WC, Kim IH (2018). Effects of different dietary n-6:n-3 PUFA ratios on growth performance, blood lipid profiles, fatty acid composition of pork, carcass traits and meat quality in finishing pigs. Ann. Anim. Sci..

[22] Ukropec J, Reseland JE, Gasperikova D (2003). The hypotriglyceridemic effect of dietary n-3 FA is associated with increased β-oxidation and reduced leptin expression. Lipids.

[23] Błaszkiewicz P, Krawiec P, Błaszkiewicz A, Pac-Kożuchowska E (2017). The role of leptin in homeostasis in the first months of life. Endokrynol. Ped..

[24] Schmitz G, Ecker J (2008). The opposing effects of n-3 and n-6 fatty acids. J. Lipid Res..

[25] Kristensen M (2012). Flaxseed dietary fibers lower cholesterol and increase fecal fat excretion, but magnitude of effect depend on food type. Nutr. Metab..

[26] Zhang W (2008). Dietary flaxseed lignan extract lowers plasma cholesterol and glucose concentrations in hypercholesterolaemic subjects. Br. J. Nutr..

[27] Kouba M, Mourot J (2011). A review of nutritional effects on fat composition of animal products with special emphasis on n-3 polyunsaturated fatty acids. Biochimie.

[28] Okrouhlá M, Stupka R, Čítek J, Šprysl M, Brzobohatý L (2013). Effect of dietary linseed supplementation on the performance, meat quality, and fatty acid profile of pigs. Czech. J. Anim. Sci..

[29] Corino C, Musella M, Mourot J (2008). Influence of extruded linseed on growth, carcass composition and meat quality of slaughtered pigs at 110 and 160 kilograms of live weight. J. Anim. Sci..

[30] Smink W, Gerrits W, Gloaguen M, Ruiter A, Van Baal J (2012). Linoleic and α-linolenic acid as precursor and inhibitor for the synthesis of long-chain polyunsaturated fatty acids in liver and brain of growing pigs. Animal.

[31] Čítek J (2015). Effects of dietary linseed and corn supplement on the fatty acid content in the pork loin and backfat tissue. Czech J. Anim. Sci..

[32] Tarricone S (2019). Effect of an extruded linseed diet on meat quality traits in Nero Lucano pigs. S. Afr. J. Anim. Sci..

[33] Bečková R, Václavková E (2010). The effect of linseed diet on carcass value traits and fatty acid composition in muscle and fat tissue of fattening pigs. Czech J. Anim. Sci..

[34] Librelotto J, Bastida S, Serrano A, Cofrades S, Jimenez-Colmenero F, Sanchez-Muniz FJ (2008). Changes in fatty acids and polar material of restructured low-fat or walnut-added steaks pan-fried in olive oil. Meat Sci..

[35] Borkowska T (2011). Influence of the addition of the linseed to fodder of slaughter animals on the content of cholesterol in the meat. Postępy Nauki i Technologii Przemysłu Rolno-Spożywczego.

[36] Ebeid T, Fayoud A, El-Soud SA, Eid Y, El-Habbak M (2011). The effect of omega-3 enriched meat production on lipid peroxidation, antioxidative status, immune response and tibia bone characteristics in Japanese quail. Czech J. Anim. Sci..

[37] Birben E, Sahiner UM, Sackesen C, Erzurum S, Kalayci O (2012). Oxidative stress and antioxidant defense. World Allergy Organ J..

[38] Shahidi F, Zhong Y (2010). Lipid oxidation and improving the oxidative stability. Chem. Soc. Rev..

[39] Min B, Ahn U (2005). Mechanism of lipid peroxidation in meat and meat products - a review. Food Sci. Biotechnol..

[40] Marcinčák S (2009). Impact of feeding of fl axseed and probiotics on meat quality and lipid oxidation process in pork during storage. Slov. Vet. Res..

[41] Oppedisano F (2020). The anti-inflammatory and antioxidant properties of n-3 PUFAs: Their role in cardiovascular protection. Biomedicines.

[42] Ahmed SM, Luo L, Namani A, Wang XJ, Tang X (2017). Nrf2 signaling pathway: Pivotal roles in inflammation. Biochim. Biophys. Acta Mol. Basis. Dis..

[43] Hu C, Yuan YV, Kitts DD (2007). Antioxidant activities of the flaxseed lignan secoisolariciresinol diglucoside, its aglycone secoisolariciresinol and the mammalian lignans enterodiol and enterolactone in vitro. Food Chem. Toxicol..

[44] Barthet VJ, Klensporf-Pawlik D, Przybylski R (2014). Antioxidant activity of flaxseed meal components. Can. J. Plant Sci..

[45] Musazadeh V (2021). Flaxseed oil supplementation augments antioxidant capacity and alleviates oxidative stress: A Systematic review and meta-analysis of randomized controlled trials. Evid. Based Complement Alternat. Med..

[46] Klimiuk K, Sembratowicz I, Czech A (2023). Effect of the inclusion of extruded flaxseed in the diet of fattening pigs on performance parameters and blood parameters. Ann. Anim. Sci..

[47] National Research Council (NRC) (2012). Nutrient requirements of swine, 11th.

[48] Friedewald WT, Levy RI, Fredrickson DS (1972). Estimation of the concentration of low-density lipoprotein cholesterol in plasma, without use of the preparative ultracentrifuge. Clin. Chem..

[49] Czech A, Sembratowicz I, Kiesz M (2021). The effects of a fermented rapeseed or/and soybean meal additive on antioxidant parameters in the blood and tissues of piglets. Animals.

[50] Folch J, Lees M, Stanley GHS (1957). A simple method for the isolation and purification of total lipids from animal tissue. J. Biol. Chem..

[51] PN-EN ISO 5509:2001. Animal and vegetable fats and oils - preparation of methyl esters of fatty acids.

[52] PN-EN ISO 5508:1996. Animal and vegetable fats and oils - analysis by gas chromatography of methyl esters of fatty acids.

[53] Weihrauch JL, Posati LP, Anderson BA, Exler J (1977). Lipid conversion factors for calculating fatty acids contents in foods. JAOCS.

[54] Ulbricht TLV, Southgate DAT (1991). Coronary disease seven dietary factors. Lancet.

[55] Fernández M, Ordóñez JA, Cambero I, Santos C, Pin C, de la Hoz L (2007). Fatty acid compositions of selected varieties of Spanish ham related to their nutritional implications. Food Chem..

[56] Statistica 10. Available online: https://www.statsoft.pl/statistica-10/ (Accessed 20 Sept 2021).

